# Modelling the innate immune system in microphysiological systems

**DOI:** 10.1039/d3lc00812f

**Published:** 2024-07-03

**Authors:** Michael J. Rupar, Hannah Hanson, Stephanie Rogers, Brianna Botlick, Steven Trimmer, James J. Hickman

**Affiliations:** a Hesperos, Inc. 12501 Research Parkway, Suite 100 Orlando FL 32826 USA jhickman@hesperosinc.com

## Abstract

This critical review aims to highlight how modeling of the immune response has adapted over time to utilize microphysiological systems. Topics covered here will discuss the integral components of the immune system in various human body systems, and how these interactions are modeled using these systems. Through the use of microphysiological systems, we have not only expanded on foundations of basic immune cell information, but have also gleaned insight on how immune cells work both independently and collaboratively within an entire human body system.

## Abstract 2

Integration of an immune component moves the field closer to a physiologically relevant human-on-a-chip.

## Graphical abstract

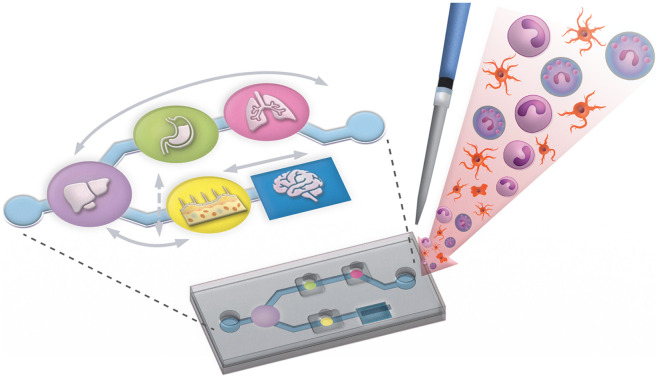

## Introducing the innate immune system

The innate immune system works tirelessly as a guardian of our health and well-being. It is our first line of defence to the world that we interact with during every moment of the day. To effectively coordinate this protection of the human body from infection many characters are at play. Together they form a network of tightly woven checks and balances to ensure the proper response is implemented to identify foreign bodies and then eliminate the harmful or disregard the commensal. While this review focuses on representation of the innate immune system (focusing on the immune cells listed in Table 1) in microphysiological systems (MPS), the innate and adaptive responses are tightly interconnected. It is necessary to discuss both to understand the direction and needs of the immunocompetent MPS field.

**Table 1:** Cells of the innate immune system

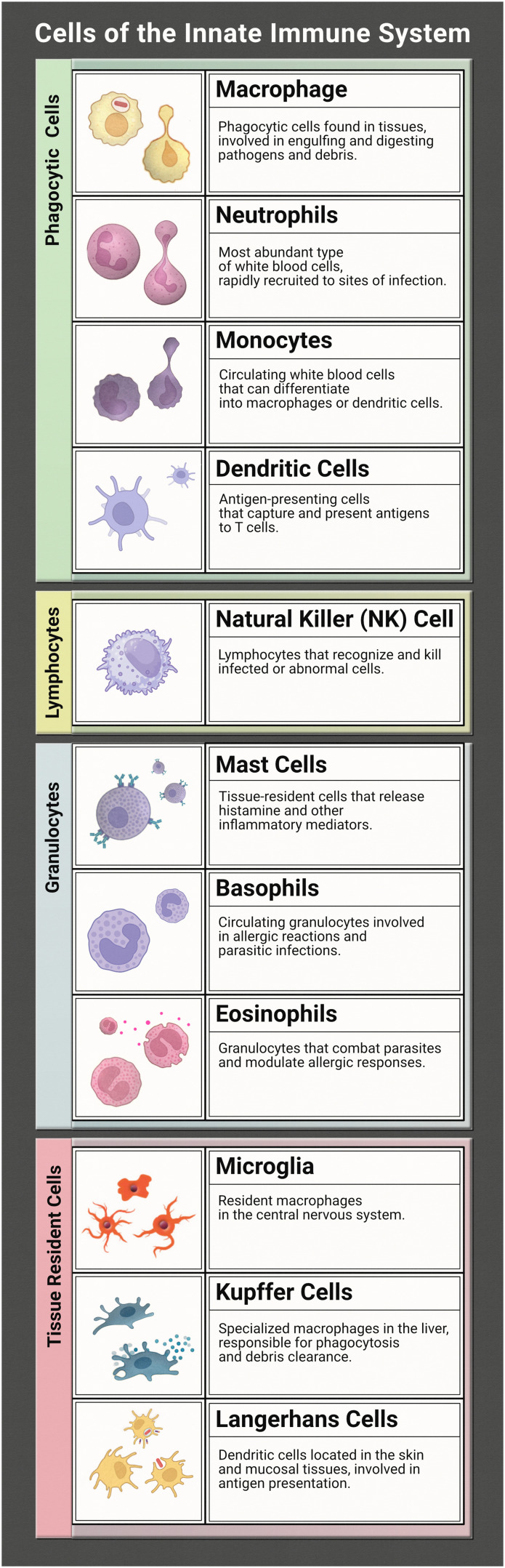

Within the human body both primary and secondary lymphoid organs reside in their specific areas with regulatory niches existing within these varying organs. The primary lymphoid organs consist of the bone marrow and the thymus, while the secondary organs consist of lymph nodes, spleen, and tonsils. The bone marrow and thymus are where immune cells migrate to mature into lymphocytes. The secondary organs are where immune cells carry out their functions during a response.^1^ The bone marrow is also the main site for haematopoiesis, or the main location where innate immune cells are produced.^2^ The unique spatial organization of the bone marrow creates the niches necessary for hematopoietic stem cells to differentiate into immune cells, with the process taking place in the spaces between reticular cells, vessels, and bone.

Recent studies suggest that infection can be detected by innate immune cells through pattern-recognition receptors (PRRs). There are three categories of PRRs: secreted, membrane bound, and phagocytic which are found in the blood stream, on cell membranes, and in the cytosol. PRRs can detect certain structures expressed by pathogens called pathogen-associated molecular patterns (PAMPs). Lipopolysaccharide (LPS) is a well-defined PAMP found on Gram-negative bacteria, another is peptidoglycan found on Gram-positive bacteria.^3^ Toll-like receptors, characteristic of a variety of hematopoietic-descended cells such as monocytes, macrophages, neutrophils, and eosinophils, are a type of PRR that can recognize many PAMPs on microbes attempting to cross cell membranes. Another type of PRR is cytosolic NLRs – nucleotide-binding domain, leucine-rich repeat proteins – which lend the immune system the ability to detect soluble intracellular pathogens. NLRs can also recognize and bind products of cellular damage such as uric acid. Binding of PRRs to PAMPs is one mechanism that spurs the immune system to mount a response against a developing infection.

Upon recognition of antigenic material, specific signalling pathways are activated depending on which PRR was bound.^4^ The exact signalling cascade may vary, but this activation generally results in release of pro-inflammatory factors such as cytokines and chemokines that in turn attract a variety of innate effector molecules such as neutrophils, macrophages, and dendritic cells to the infection site. Dendritic cells are especially important at this point, as they will take up antigen, become activated and then deliver the antigen to the lymph nodes to assist in the development of an adaptive response. The influx of effector molecules also allows important innate responses such as phagocytosis of pathogenic material to begin, most swiftly by neutrophils as well as macrophages and granulocytes.

Neutrophils are commonly understood to be first responders of the immune system and as such they are fully equipped to handle an array of pathogens. Common inflammatory responses include producing reactive oxygen species to inflict damage to the pathogen, deploying neutrophil extracellular traps (NETs) to snare them, ingesting them through phagocytosis, and even signalling other immune cells for support.^5^ Neutrophils release inflammatory cytokines to recruit antigen presenting cells (APC) such as macrophages. This in turn results in the release of more inflammatory cytokines that signal further recruitment of neutrophils, thus generating an inflammatory feedback loop in an acute inflammatory process known as neutrophil swarming.^6–8^ Recruited innate cells will then utilize their specialized antigen presenting skills by ingesting pathogenic material and delivering it to components of the adaptive immune system for further action.

This of course is an oversimplification of the innate immune process and still there are many other moving parts involved. There are many diseases linked to the immune system, plus immune response to clinical therapies can greatly influence therapeutic efficacy, so it is imperative to understand this network of interactions. Although a therapeutic compound is highly regulated as it progresses through simple, *in vitro* single-organ studies and animal models, it may still fail when it is finally tested in human trials. While safeguards are in place, failures are common as the prior tests lack the complexity of the human body – especially the intricate machinations of the immune system. Integrating MPS into this framework could help to reduce economic burden, adverse effects in humans, and the number of animals used in therapeutic development.

## Modelling primary immune tissues

MPS are still a relatively novel design that has become a rapidly growing field. Following quickly on the heels of microfluidics developed in the 2000s, we see the appearance of microphysiological platforms for the use of cell culture take off around 2012.^9^ The last decade has yielded a multitude of various organ-on-a-chip models that have contributed to the biomedical field. As we continue to advance, there is an understanding that the need for models of the immune system are necessary. There are many organ-on-a-chip models that exist to physiologically mimic immune organs (*e.g.*, bone marrow, thymus, lymph nodes, spleen). Models recapitulating the human bone marrow (hBM) tend to be more focused on the innate immune component while others, like lymph nodes and thymus, are centred more on the adaptive immune system – more specifically on T lymphocytes.

The human bone marrow (hBM) is a complex organ essential to the immune system and is critical for both hematopoietic and immune homeostasis. Nelson *et al.* designed a hBM-on-a-chip model with a 96-well format, which allows for eight independent “chips” to be studied at once, which is not typically seen in organ-on-a-chip models.^10^ This model was designed specifically so that endosteal, central marrow, and perivascular niches of the human bone marrow could all be incorporated. The composition and microphysiology of hBM is accurately mimicked in this complex multi-niche microtissue due to how various cell types are differentiated and seeded into the model. To mimic the bone-like endosteal layer of hBM within the model, osteogenic differentiation of human mesenchymal stromal cells (MSCs) was performed, which produced mineralization of the bottom surface of the device. Once this endosteal layer was in place within the hBM-on-a-chip, human endothelial cells and MSCs were seeded in a fibrin-collagen hydrogel network (mimicking the central marrow), creating a 3D microvascular network, which mimics the perivascular niche of the hBM. Nelson *et al.* discovered one of the key insights that this model provided was that the presence of the endosteal niche caused a decrease in proliferation and increased maintenance of CD34+ hematopoietic stem cells. The hBM is considered an immune-privileged site for hematopoietic stem cells, which are responsible for giving rise to a variety of immune cells through differentiation.^11^

Another model that recapitulates the key physiological properties of the hBM microenvironment was designed by Cairns *et al.* They designed their model, known as the “Humimic Chip2”, to observe bone marrow toxicity as a side effect of treatment with oncology drugs, which is a phenomenon that directly affects the immune system.^12^ The specifications of this “Humimic Chip2” include a 3D ceramic scaffold (made from hydroxyapatite) with a pore size and structure within the device that mimics the human cancellous bone. CD34+ human stem and progenitor cells (HSPCs) were co-cultured with MSCs to provide the porous environment present in hBM, and medium was circulated *via* a microfluidic channel to maintain the simulation of the bone marrow niche. The use of MSCs in this model is extremely important because they have the capacity to induce immunomodulation of varying cell types involved in both the innate and adaptive immune responses.^13^ The only way to assess the hBM toxicity within this model is to analyse the hBM through an endpoint assay. The specific endpoint assay used by Cairns *et al.* was flow cytometry, achieved by staining the cell populations within the medium and the 3D scaffold of this model and looking to see which types of immune cells were still present after dosing the system with various drugs. The “Humimic Chip2” model gives researchers the baseline they need when it comes to designing models for drug development regarding the bone marrow.

Within all the lymphoid organs, mature innate immune cells, most commonly macrophages and DCs, cause an initial innate response to foreign cells within the body. Upon activation these antigen presenting cells will travel to secondary immune organs, such as lymph nodes, to activate an adaptive immune response. Human lymph nodes (hLN) are bean-shaped structures that line lymphatic vessels acting as filters, where immune cells trap germs and stimulate the production of antigen-specific antibodies *via* adaptive immune processes.^14^ hLNs play a crucial role in the systemic circulation of the body. At the thoracic duct, lymphatic vessels return lymph and its content to blood.^15^ In a hematogenous context, propulsive lymph flow drives metastasis in lymph-borne cancer cells, as hLNs are integral in the spread of cancer into circulation.^16^

Shanti *et al.* wanted to design a microenvironment that simulated the hLN to work on drug development.^17^ They produced a LN-on-a-chip model where they inserted different types of immune cells (dendritic cells and macrophages) into the microfluidic device to incorporate key features of the hLN. These characteristics entail similarity in the components of the extracellular matrix (ECM), replication of lymphatic flow pattern, viability of cells encapsulated in collagen throughout the duration of immunotoxicity experiments, compartmentalization of immune cells, and interactions of different cell types across the different chambers on the system. By creating these features, they demonstrated that this LN-on-a-chip model can maintain cell viability for more than 72 hours, which is the time necessary to study drug toxicity. Future models regarding the hLN could be designed to investigate the role of innate immune cells in more detail. Elevation of a hLN microphysiological platform could offer insights to effects on migration of innate APCs and the interaction leading to the activation of adaptive immune cells in response to drug therapies or various disease phenotypes.

The thymus, tonsils, and spleen all appear to be underrepresented in organ-on-a-chip models up until this point, with these organs being focus points on the development of new immune response models. The thymus is a gland-like organ where, as mentioned before, T cells mature into lymphocytes, and it is responsible for coordinating the processes between the innate and adaptive immune systems. The tonsils consist of three different regions: palatine, lingual, and adenoids, all containing white blood cells responsible for killing germs or starting an immune reaction. The spleen is one of the more complex lymphoid organs with functions ranging from breaking down erythrocytes, storing and breaking down thrombocytes, and storing various immune cells such as phagocytes, which act as a filter for germs that get into the bloodstream.^1^ Incorporating these three lymphoid organs into future MPS designs would help researchers paint the full picture of how the immune system responds to different stimuli.

Over the last decade vast strides in the development of MPS have benefited our understanding of organ-to-organ interactions in a laboratory setting. As these systems have grown in complexity there has been an understanding that the immune system must be integrated into these platforms. In this review we will continue to cover the development of the innate immune system in the realm of MPS, especially as it pertains to the incorporation of innate immunity into models of various body systems.

## The innate immune system and the epithelium

Epithelial linings from our skin to the walls of our gut help to deter any opportunistic pathogens that seek to cause us harm. This serves as a physical barrier to the vulnerable tissues and provides a home for harmless bacteria that prevent the colonization of these pathogens. The flora that resides on each of us is unique and is known as our microbiome. The microbiome is an intriguing topic that has garnered much interest in recent years, with many studies finding various diseases linked to the dysbiosis of commensal bacteria found on and within the human body. When reviewing the innate immune system, there are many nuances to address regarding the relationships between our immune cells, the epithelium, and the specific microflora crowding our epithelial tissues.

**Fig. 1:**
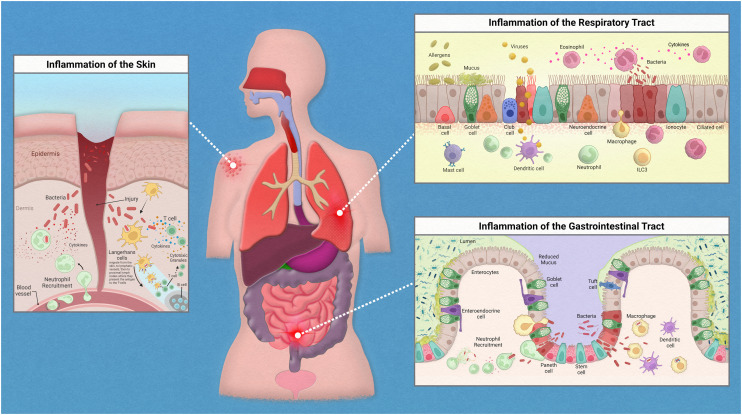
Inflammation in epithelial tissues.

While the inflammatory process in the human body has similar key players involved throughout, the exact mechanism of action is regionally specific for each organ (Fig. 1). The epithelium of the skin consists primarily of stratified keratinocytes with Langerhans cells interspersed throughout them. Under homeostatic conditions these cells work together to maintain barrier integrity and to distinguish between commensal and pathogenic microorganisms to prevent infiltration of infectious agents. An inflammatory response could be initiated due to direct injury that then allows opportunistic pathogens direct access to our circulation. Langerhans cells, which are dendritic-like cells, capture pathogenic material, and can migrate through the tissue to nearby lymph nodes to present the antigen to adaptive immune cells. Inflammatory cytokines released by keratinocytes recruit neutrophils from the peripheral blood to the site of injury.

The epithelium of the gastrointestinal tract consists of enterocytes, enteroendocrine cells, goblet cells, intestinal stem cells, Paneth cells, and tuft cells. Under homeostatic conditions these cells work similarly to that of the lung in the sense that they form both a physical barrier and mucosal barrier to microorganisms. Tight junctions of the epithelium form a strong barrier that is protected by a layer of antimicrobial mucous attributed to Paneth and goblet cells. Inflammation in the gastrointestinal tract could be attributed to food allergies or a variety of opportunistic pathogens. Common progression of inflammation in the gut includes degradation of the mucus layer allowing opportunistic pathogens access to epithelium. Thus, resulting in similarly described immune cell recruitment and antigen presentation.

The epithelium of the respiratory tract consists of basal cells, ciliated epithelial cells, club cells, goblet cells, ionocyte, and neuroendocrine cells. In healthy lung tissue, basal cells generate ciliated and secretory cell types, epithelial cells maintain barrier integrity, and the goblet and club cells secrete antimicrobial mucins. Inflammation in the respiratory tract could be attributed to a variety of stimuli such as allergens, bacterial infection, or viral infections. Common immune response include increase in antimicrobial mucous production to trap pathogenic material that can then be swept away by ciliated epithelial cells, release of inflammatory cytokines to attract neutrophils and macrophages, followed by phagocytosis and antigen presentation to adaptive immune cells.

### The skin

The skin, the first line of defense against many of the pathogens encountered daily, is a prominent place to start when we think about modeling immune interactions with the epithelium. MPS of the skin are one of the most vastly used throughout the industry – fitting as the skin is also the largest organ. With the growing pressure exerted on the cosmetics industry to move away from animal testing, modeling skin using MPS is a necessary alternative. The skin provides a prime model to study the immune system as it encounters many irritants, whether it be a hypersensitivity or an allergic reaction to a product, damage due to UV exposure, or even direct insults that cause damage to the epithelial barrier. All of these may elicit a different form of immune response, which makes the field of MPS skin modeling diverse.

Ramadan *et al.* delivered a MPS for monitoring interactions between the skin and immune cells.^18^ For this model they established a co-culture of keratinocytes and DCs to investigate protective qualities of the skin by exposing keratinocytes to UV. They also monitored immune responses of the DCs when their model was exposed to infection (through exposure to LPS) in the presence or absence of keratinocytes. Certainly, to no surprise, they found that UV exposure degrades keratinocytes and weakens their tight junctions. They also found that immune responses were dampened with the protective barrier provided by the keratinocytes. Their model was not without limitations, as the immune cells used were an immortalized line and do not behave the same as primary human immune cells, however they offered a scaffold from which more researchers could build.

Work that explores this interaction a little deeper was presented by researchers Chau *et al.* when they introduced a 3D immunocompetent model of the skin in early 2013.^19^ Their multi-layered model incorporated an air–liquid interface (ALI) with an optimized medium to support the culture of keratinocytes, fibroblasts, and DCs. All of the cells utilized in this platform were acquired from primary human donors. DCS sandwiched between the epidermal keratinocytes and lower dermal fibroblasts remained viable for 5 days and were able to successfully migrate through the layers and out into the liquid layer of the model while retaining their viability. They were then able to administer a skin sensitizer, dinitrochlorobenzene (DNCB), to the apical surface and monitor immune response. They found that upon application of DNCB a significant increase in DCs migrated through the layers of the skin. There was also an upregulation of CD86 on the DCs, which is utilized for antigen presentation and T-cell activation.

Researchers Kwak *et al.* further expanded on the modeling of the immune response in the skin by addition of a vasculature component to their model.^20^ The skin is highly vascularized and relies on this network of microvessels to maintain homeostasis. The ability to incorporate a vascular component to the skin allows exploration of avenues of nutrient delivery, wound healing, and immune response to infection. Their tripartite skin model (consisting of a human keratinocyte cell line, primary human fibroblasts, and HUVECS) worked to recapitulate the epidermal, dermal, and endothelial layers of the skin. Their system was also subjected to gravity driven flow to mimic the fluidic condition of the vasculature of the skin. They found that their model could elicit an inflammatory response when subjected to SDS, a known skin irritant. A significant increase in IL-6 production was observed when exposed to sodium dodecyl sulfate (SDS) compared to unexposed controls. They further added to this skin model by integrating leukocytes to study immune migration in response to injury. Upon exposure of the skin model to UV, they were able to capture trafficking of the leukocytes from the vasculature component to the site of the injury.

### The gastrointestinal tract

As we further explore modeling of the innate responses and epithelium, a focus to microfluidic devices of the gut and their incorporated immune cells will be reported. Resident immune cells in the gut, primarily consisting of macrophages and DCs, have the important job of discerning when to initiate an inflammatory immune response and when to proceed as usual. When these immune cells encounter pathogenic material in circulation or in other tissues, the innate response eliminates the pathogen. However, with the high population of commensal microbiota found within the gastrointestinal tract, if immune cells employed an inflammatory response during each interaction there would be sustained, chronic inflammation. Oftentimes this results in what is known as inflammatory bowel disease. Instead, these resident immune cells should specifically target foreign opportunistic pathogens before they can disseminate disease throughout the body.

Many developments in intestinal modeling have been made, however many have excluded the immune perspective. In 2010, researchers Leonard *et al.* recognized the need for inclusion of an immune component to fully represent disease conditions of the gastrointestinal tract.^21^ A trans-well-based triculture of Caco-2 cells with primary human blood derived macrophages and DCs were used to monitor responses when inoculated with LPS or treated with pro-inflammatory cytokines. Upon encountering the pro-inflammatory stimuli, the following occurred: increased disruption in the epithelial tight junctions, increase in transportation across the membrane, and increase in pro-inflammatory markers – all of which were further increased when compared to a monoculture consisting of only Caco-2 cells. Additionally, migration of immune cells to the apical side of the model and uptake of particles was observed during the inflammatory response. This work points out the necessity of the immune components when modeling interactions that take place within the gut.

As the importance of the gut microbiome became clearer in the proceeding years, researchers investigated ways to model these interactions in a human-based, physiologically relevant manner. A microfluidic interface was created in 2016 that modeled interactions between human and microbials in the gastrointestinal tract called HuMiX.^22^ This interface, established by researchers Shah *et al.*, created microenvironments within the model consisting of various oxygen concentrations for the co-culture of human cells and bacteria. Immunological responses of gut epithelial Caco-2 cells were observed when co-cultured with gut bacteria *Lactobacillus rhamnosus* GG. This co-culture suggested an anti-inflammatory response to the bacteria as there were decreases in the cytokine detection of CCL20 and IL-8. Additionally, while not the primary focus of the work, preliminary studies also determined the capability of inclusion of CD4+ T-cells to model immune response.

An exciting and more recent development in modeling the human intestinal environment was recently demonstrated by researchers Maurer *et al.* This microfluidic biochip recapitulated endothelial and epithelial gut components co-cultured with peripheral blood mononuclear cells (PBMCs) that were differentiated to represent mucosal macrophages and dendritic cells.^23^ As both immune cells are integral in maintaining the homeostatic conditions of the gastrointestinal tract, this model provides useful insight to the modeling of the gut. Moreover, immune mediation to gut microbiota was observed with the introduction of microbial associated molecular patterns (MAMPs) in the form of LPS and from commensal and opportunistic microbiota such as *L. rhamnosus* and *C. albicans*. Primary HUVECS and human epithelial colorectal cell line Caco-2 cells were cultured on opposing sides of a porous membrane to simulate an intestinal vasculature (endothelial side) and an intestinal lumen (epithelial side). They found that exposure to LPS on the endothelial side resulted in a pro-inflammatory response and disruption to the barrier integrity, as expected in cases of endotoxemia. In contrast, no inflammatory response was observed when exposed to LPS on the epithelial side. Further experimentation demonstrated the benefit of commensal microbiota. Colonization of *L. rhamnosus* on the epithelial side not only failed to stimulate a pro-inflammatory immune response but contributed to the protection of barrier integrity during LPS infection. Additionally, this colonization inhibited the growth of opportunistic pathogen *C. albicans*. This model offered exciting new insights in modeling the complex nature of tissue specific immune cells as we know their behavior is vastly different from those in systemic circulation. The capability to model immunotolerance of certain microbiota while also observing inflammatory responses to pathogens, as noted in this study, is necessary to fully understand the complex machinations of the gut.

### The respiratory tract

The need for models to study the respiratory system, especially as it pertains to immune response, has become increasingly apparent since the global pandemic resulting from SARS-CoV-2. We all now understand the gravity of the matter and the use of MPS can help to fortify our understanding of respiratory illnesses.

In 2010, researchers Huh *et al.* recapitulated the lung by developing an alveolar-capillary interface to study neutrophil response to pro-inflammatory stimuli.^24^ When introducing tumor necrosis factor alpha (TNF-α) in the medium, characteristic lung inflammation responses were observed such as: increased ICAM-1 expression, binding of neutrophils to ICAM-1, and transmigration of the neutrophils across the capillary-alveolar membrane. Response to bacterial infection could also be modeled using this platform. When the alveolar microchannels were inoculated with *Escherichia coli* bacteria, primary human pulmonary microvascular endothelial cells were activated and in response neutrophils extravasated across the barrier toward the infection, resulting in phagocytosis of the bacteria.

In 2015, researchers Benam *et al.* effectively modeled a small airway-on-a-chip to study inflammatory responses in the lung.^25^ This model consisted of both the epithelial and endothelial components to mimic human bronchioles allowing for phenotypic disease models of asthma and chronic obstructive pulmonary disease (COPD). Primary human airway epithelial cells were exposed to air while primary human pulmonary microvascular endothelial cells were exposed to medium. These cell types were seeded on opposite sides of the same porous membrane to allow for cell-to-cell interaction. When the model was exposed to IL-13, asthmatic conditions were induced, resulting in increased goblet cells and inflammatory cytokines coupled with a decrease in ciliary beat frequency.

Childhood asthma can develop into two different phenotypes known as non-allergic (eosinophilic) and allergic asthma, with the base immune mechanism being relatively the same.^26,27^ Both types are driven by type 2 inflammation, characterized by airway inflammation resulting in both airway mucus plugging and bronchoconstriction.^28^ Not only does dysfunction within the innate immune system lead to childhood asthma development, but adult asthmatics experience exacerbations due to primary immunodeficiency, which causes recurrent infections in both the upper and lower respiratory tracts.^29^

To study inflammatory responses in patients with asthma or COPD, the same model by Benam *et al.* was exposed to polyinosinic–polycytidylic acid (p(I:C)), which functioned as a mimic of viral infection. For the asthmatic model, this exposure not only resulted in a pro-inflammatory response in the epithelium (marked by an increase of RANTES and IL-6), but also an increase in endothelial adhesion markers resulted in increased recruitment of neutrophils. To model COPD healthy epithelial cells were replaced with diseased COPD epithelial cells. For the COPD model, systems were either exposed to LPS to induce macrophage activity or p(I:C) to induce neutrophil activity. Both of which generated a pro-inflammatory response, however only the LPS-treated systems saw a significant increase in pro-inflammatory cell response when compared to healthy controls. The increase in GM-CSF, a macrophage stimulating factor, suggests that it may require more attention when studying infection in COPD patients.

Further studies of viral activity in the lungs were published in 2021 by Si *et al.*^30^ A microfluidic bronchial-airway-on-a-chip system that is lined by both pulmonary and bronchial-airway endothelium was used for the purpose of rapidly identifying candidate therapeutics for varying diseases and viruses that affect the lungs. Within this model, it was also possible to demonstrate the recruitment of immune cells as part of a response. Upon infection, primary neutrophils migrated to the infected chip within the system through vascular channels and recruited even more circulating immune cells to the apical surface of the lung epithelium.

Chen *et al.* created a lung-on-a-chip platform that contained alveolar and bronchial components, resident macrophages, and circulating monocytes.^31^ Within this model, they constructed and used a fully automated spreading system capable of simulating the advancement of infection within the lung. Once infected, they were able to analyze and classify both the inflammation-induced changes in the lung epithelium and varying macrophage morphology, due to a deep-learning-based recognition module included within the system. Not only is an inflammatory immune response induced upon infection, but it is also induced upon injury to the human body, so it is imperative that future organ-on-a-chip models are designed with a component to simulate cell injury to observe and analyze the response.

As we contemplate improvements to respiratory MPS, it's important to also consider the unexplored microbial terrain of the lungs. When most think about the microbiome common things that come to mind would be the skin or the gut, but what about the lungs? While the lungs contain a million bacteria per gram of tissue less than that seen in the gut,^32^ they still influence our body's responses in healthy and diseased states. Clinical perturbations in the lung microbiome has been studied in relation to asthma,^33^ COPD,^34^ cystic fibrosis,^35^ and other respiratory diseases. Respiratory MPS offer novel platforms to dissect the balance between commensal and opportunistic microbes in airway homeostasis.

## Migration models of the innate immune system

**Fig. 2:**
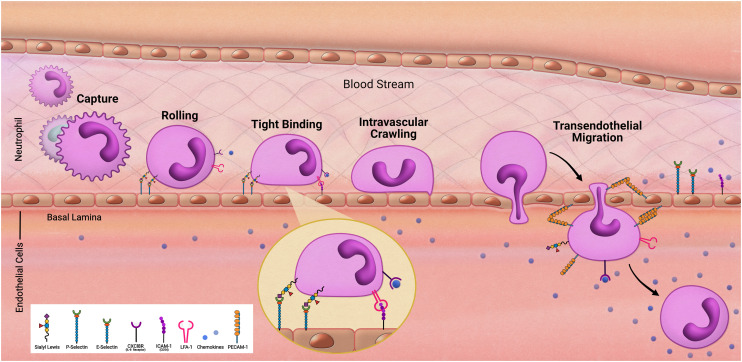
Transendothelial migration of neutrophils.

As we follow our immune response from the outer walls of the epithelium to our blood and tissues it will be important to understand how these cells arrive at these locations. As development of the innate immune system using microfluidic devices has advanced, a primary focus has been on modeling inflammation. Replicating the trafficking patterns of leukocytes is an integral component in our understanding of inflammatory pathways. Systemic immune cells circulate throughout our periphery and extravasate into our tissues when summoned (Fig. 2). This migration into the tissues is mediated by rolling adhesion as the immune cell begins to bind to epithelial markers such as ICAM. As the immune cell comes to a rest, it is removed from the bloodstream as it squeezes in between the vascular endothelial wall and infiltrates our tissues. To properly model such aspects of immune recruitment, fluidic conditions are required. Complex systems such as animal models have contributed to this, however due to inconsistencies between humans and animals, animal models can only enlighten our understanding so much. For example, neutrophils make up approximately 60% of circulating leukocytes in humans but only approximately 18% in mice.^36^ Through the use of microfluidic devices in a study by Jones *et al.* it was also uncovered that, in response to chemoattractants, activation and migration of human neutrophils are significantly higher than rodent neutrophils.^8^ Complex *in vitro* models such as MPS allow for the incorporation of immune cells under microfluidic conditions that cannot be obtained under traditional cell culture methods. By demonstrating the success in establishing trafficking patterns of immunocompetent MPS, which is necessary when monitoring immunotoxicity, the field can offer an alternative to animal models for preclinical studies.

Researchers Jones *et al.* in 2012 developed microfluidic chambers to study trafficking interactions among a heterogeneous population of neutrophils and monocytes.^37^ This device consisted of a loading chamber with several microfluidic channels, some of which would lead to chambers pretreated with chemoattractant. Initially, observations found that neutrophils arrived the fastest with monocytes arriving hours behind, reflecting *in vivo* characterization of neutrophils as first responders. Interestingly, subpopulations of monocytes were determined based on travel time and found that monocytes with high elastase activity, a phenotype of activation in response to inflammation, arrived faster than those that did not have elevated elastase activity.

In a study performed by Hamza *et al.* in 2015 a microfluidic device was developed to monitor the trafficking of neutrophils in response to stimuli.^38^ The loading of a single drop of whole blood into this platform allowed for subsequent migration of neutrophils out of the loading compartment while retaining erythrocytes. Zymogen particles were added to compartments surrounding this chamber and leukotriene B4 (LTB4) was utilized to promote chemotaxis of the neutrophils from the central compartment. This study illuminated the self-regulating response of neutrophils by monitoring their flow to the site of infection, as well as their return to the central compartment. It was observed that neutrophils that phagocytosed the pathogenic material remained in the external compartments. However, neutrophils that did not interact with the zymogen particles returned to the central compartment. This provided a microfluidic approach to understanding how the innate immune response can mitigate infection without inducing chronic inflammation that would lead to tissue damage.

Migratory habits of innate immune cells do not just include traversal to the site of infection. Antigen presenting cells, such as DCs, must also navigate to immune organs such as lymph nodes to alert and activate the adaptive immune response. Researchers Mitra *et al.* sought to model this interaction in 2013.^39^ Their model consisted of fluidic pathways with a CCL-19 chemokine gradient that should direct DCs to a T-cell chamber and result in T-cell activation. Access to the chemotaxis channels would be barred to inactivated DCs based on their morphology. However, they observed morphological changes upon activation, transitioning from a circular to an amoeba-like shape, allowing for migration into the chemotaxis channel. DCs following the chemokine gradient would then land in the T-cell chamber and induce T-cell activation.

In 2020 a study performed by Sasserath *et al.* integrated THP-1 cells to a five-compartment microfluidic platform containing three different organ constructs: heart, liver, and skeletal muscle.^40^ This platform was maintained over the course of 7 days to model innate immune response activation pathways. Cardiotoxin amiodarone was administered to monitor a tissue specific infiltration of immune cells. M2 activation was observed in the system as a response to cardiac damage and infiltration of the cardiac cells occurred while no presence of the THP-1 cells was detected in the skeletal or hepatic components - additionally, no effects on hepatic or skeletal muscle viability or functionality was observed. However, upon delivery of LPS and INF-γ, M1 activation was observed and as a result effects of a pro-inflammatory immune response could be seen across all organ constructs. This work elucidated the opportunity to model immune response in human-on-a-chip models by demonstrating activation pathways of recirculating immune cells in a multi-organ construct.

The ability to capture how immune cells respond to stimuli is incredibly useful to model in a human-based platform. Oftentimes in animal models they must be immunocompromised to study human cellular components of interest. These researchers have supplied the field with novel opportunities to investigate immune response, both in their ability to locate antigens and then navigate to a secondary location to present antigens to adaptive immune cells.

## The innate immune system and the central nervous system

Maintaining functional immunity is especially important to the physiology of the central nervous system (CNS), as the brain and spinal cord are responsible for maintenance and coordination of every bodily function. As such, including immune components in microphysiological models of the CNS is vital to generating complete disease models.

The resident immune cells of the brain include microglia and astrocytes. These perform constant immunosurveillance and respond to pathogens and injury by attracting and emitting effector molecules.^41^ One notable attribute of these cells is their adaptability; microglia and astrocytes exhibit a wide range of functions in their immune responses within the central nervous system.^42^ Microglia maintain homeostasis in the brain, with heterogeneous roles that coincide with brain locale. Some diverse functions include phagocytosis, regulation of synapse health, and stimulation of neuron proliferation by secretion of growth factors.^43^

Regarding their immune function within the brain, microglia become activated following injury or detection of pathogens by their mobile processes. Threat identification results in phenotype switching specific to the issue to which the cell is exposed. Microglial functions may delay or aggravate disease progression, depending on the disease stage and balance of pro- and anti-inflammatory factors. Further studies are necessary to determine the paths to different effects and how microglial responses contrast in distinct disease states.^44^ Including microglia in microphysiological models could help determine specific instances in which microglial activation hinders recovery and could be useful to developing therapeutics that target harmful glial activity.

Astrocytes are specialized in blood–brain barrier (BBB) maintenance, balancing cerebral pH, water content, and blood flow. These cells also release anti-inflammatory cytokines under normal conditions and are efficient at detoxifying glutamate – a neurotransmitter that can become harmful at high concentrations.^45^ Like microglia, astrocytes can adopt reactive phenotypes that inhibit restoration of health.^46^ Consequently, including astrocytes in models to observe their effects on neuronal disease progression is crucial to identifying novel therapeutic targets to slow or reverse degeneration.

**Fig. 3:**
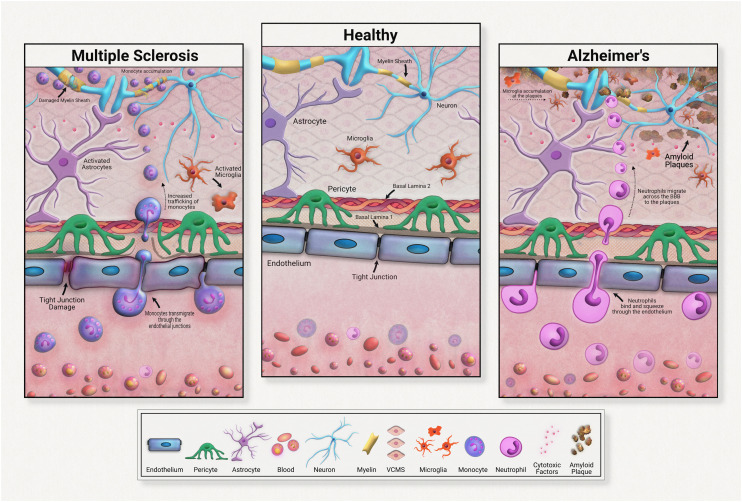
Neuroinflammation and the blood–brain barrier.

Although all these immune cells normally regulate the brain's stasis, their negative impacts and contributions to disease development must be acknowledged and investigated. Immune cells are heavily implicated in chronic neurodegeneration (Fig. 3). Maintaining a healthy blood brain barrier is integral to homeostasis within the central nervous system. As the resident immune components of the brain, microglia and astrocytes are responsible for upkeep of neural immunity. Microglia are known for their pathogen detection skills and subsequent cytokine release; astrocytes are also known for their cytokine release, especially anti-inflammatory subtypes. Both of these cell types are highly heterogeneous in their effects, meaning that they have a wide range of outputs that depend on the disease or pathogen detected. In many diseases, these cells further pathology, commonly through inflammation. These immune cells can become defective, emitting an excess of pro-inflammatory factors. These factors contribute to tissue degradation and also attract other immune molecules across the blood brain barrier, further compounding inflammation.

For example, in Alzheimer's disease (AD), microglia exposed to developing amyloid beta plaques will eventually release excessive proinflammatory factors that exacerbate disease progression.^48^ Upon exposure to developing amyloid-beta plaques, microglia release cytokines that create an inflammatory state and attract neutrophils across the blood brain barrier. Migrated neutrophils then release NETs and IL-17 in the brain, accelerating neuroinflammation. Although these immune cells are normally beneficial to health, their defective natures must be further investigated in the development of therapeutic strategies.

Neuroinflammation in multiple sclerosis is caused in part by excessive monocyte migration across the BBB as a result of astrocyte dysfunction.^49^ Astrocytes lose their glutamate detoxification skills, resulting in buildup that leads to neural tissue damage. Monocytes, a normally peripherally circulating cell population, are attracted across the blood brain barrier in MS, accumulating at the neurons. These can also undergo differentiation to inflammatory macrophages, releasing ROS and NO that causes tissue damage. In a mouse model of multiple sclerosis, chronic inflammation was associated with astrocyte release of lactosylceramide, which caused a signal chain resulting in further inflammation and neurodegeneration.^50^

Early CNS *in vitro* models focused on characterizing the growth of neurons and neuronal stem cell progenitors.^51,52^ The effects of different cell factors, microenvironments, and therapeutics on cellular processes such as neuronal proliferation, differentiation, and myelination were also explored in early models.^53,54^ As early systems were naturally simpler and often monocellular, they were devoid of glia or other immune cells and did not demonstrate interactions observed *in vivo*. The necessity of including immune cells in brain MPS became more indisputable as evidence of the role of immune responses in neurodegenerative diseases emerged, as reviewed by many.^55–58^

An initial model containing central nervous system axons and one glia type, oligodendrocytes, was fabricated by Taylor *et al.* in 2005.^59^ This platform consisted of two compartments: a large well that allowed for the growth of soma, and narrow tunnels that allowed for CNS axon isolation and addition of oligodendrocytes. Microgrooves on the chamber surface enabled manipulation of the direction of axon growth. Isolating axons from soma allowed for studying glia–axon interactions and effects on myelination. Changes in gene expression at the soma due to axon manipulation were also examined. This model was especially important to the early development of CNS MPS because it indicated that axonal growth paths could be manipulated without compounds. Directing growth by manipulating the physical paths of the microfluidic chamber reduced the confounding factors compared to using chemical means. The isolation of soma from the axons and glia allowed for more individualized investigation of how manipulation at either locale affected overall neuron health. Although it is not specifically known as an immune cell, the inclusion of the oligodendrocyte here represented an early effort towards the determination of other CNS cells' impact on neural health. Notably, this model was valuable in its use of CNS axons rather than those of the peripheral nervous system, overcoming the difficulties associated with culturing central nervous system cells. A caveat of this accomplishment is that the cells were rat-derived; this model would be even more applicable to human biology if human cells had been incorporated.

Majumdar *et al.* developed a platform that allowed for further manipulation of individual cellular environments in 2011.^60^ Their platform included a valve that controlled exposure of neuron cultures in one chamber to glia in the other, allowing for individual manipulation of each cell type. These researchers were able to deduce an improvement in neuron viability in the presence of glia; neurons cultured alone in the system could not survive for longer than a week. In addition to this viability improvement, neuron–glia co-cultures in the MPS demonstrated a 3–4-fold increase in transfection efficiency when compared to traditional culture methods on plates. This system proved the importance of including glia in MPS by displaying the increased viability and transfection capability of neurons cultured in their presence. Administration of drugs at either chamber allowed for observation of effects on the treated cell type as well as the adjacent one upon opening of the valve. Developing a system that allowed for controlled exposure of neurons to glia demonstrated the shifting focus of the industry towards gleaning immune cell interactions within the central nervous system. As this study used rat-derived neurons and glia, it would be valuable to repeat these experiments with use of human cells to determine if the same effects on neuronal performance are present. Characterization of the glial cell population distribution would also be an improvement here since different glia types have separate roles in brain immune regulation.

More specific systems that utilize a single glia population, such as microglia, would be useful for characterizing individual cell types and their range of effects. It is now recognized that several microglia subtypes with various capabilities are found within the CNS.^61^ Subtypes have been proposed with roles in surveillance, pruning, phagocytosis and neuromodulation of synapses.^62^ As discussed above, despite their host of beneficial effects within the CNS, microglia are also implicated in the inflammatory pathology of neurodegenerative disorders such as AD, making them an attractive candidate for therapeutics.^63^ AD is characterized by a progressive decline in cognitive and synaptic function, caused by amyloid-beta plaque deposits and neuroinflammation. This inflammation is theorized to be caused by neutrophils, which migrate to the brain and release NETs and IL-17, demonstrating a significant role of these in neurodegeneration.^64^

To determine the role of microglia in this process, in 2019 Park *et al.* devised a microfluidic system that included microglia and neutrophils and assessed their interactions in the presence of amyloid-beta.^65^ The model's use of human cell lines exemplified the field's shifting focus toward higher applicability. This system consisted of two compartments: a central circular well that contained microglia and soluble amyloid-beta, and a second compartment surrounding the inner one that held isolated neutrophils in suspension. The two compartments were connected by microchannels through which only the neutrophils could pass. When assessed, inflammatory cytokine expression was upregulated in amyloid-exposed microglia. After neutrophils were added, migration of those towards the microglia-rich center was observed. Various cell densities were used, and it was found that neutrophil migration increased in proportion to microglia density. Additionally, two pro-inflammatory factors, IL-2, and MIF, were found to be released in the microglia–neutrophil co-cultures. This further demonstrated the system's functionality as these factors are known to increase in AD patients.^66,67^ This system capably exhibited the chemoattractant nature of inflammatory neural microglia and, in the future, could provide insight into the effect of novel therapeutics on this process. An interesting extension of this work could be to include neurons in the model to assess cell interactions in the presence of amyloid-beta.

Aging related neurological dysfunction has been theorized to be caused by continuous low-grade inflammation in the brain that slowly wears down health.^68^ This low-grade inflammation may be offset by anti-inflammatory factors, but in diseased states, the pro-inflammatory factors outweigh this balancing attempt. An inherent advantage of MPS is their ability to customize the origin of the cells used in modelling. Cells could be derived from people with different physical characteristics to determine if readouts or cell activity differs between the two. As aging itself is a risk factor for a variety of neurological disorders,^69^ investigating the differences between young and aging patients' immune cells is important to developing models that accurately represent older populations.

To further research for the inflammation common in older patients, Ao *et al.* in 2022 devised a microphysiological device that allowed for interactions between monocytes and cortical organoids.^70^ Monocytes were of interest to these researchers as they are theorized to contribute to ‘inflammaging’, inflammation that increases with age as a result of leaky tight junctions and upregulated adhesion molecules on the blood brain barrier. Once across the BBB, it is thought that these cells become activated and release an excessive number of inflammatory factors such as TNF-α. Monocytes were derived from PBMCs of young, 20 to 30 year-old donors as well as older, 60+ year old donors. Cortical organoids were grown underneath mesh scaffolding that allowed for monocyte-containing medium to flow through. After addition of monocytes, the device was placed on a rocker to induce flow and cell–cell interactions. Neuron morphology and the presence of inflammatory and aging-related markers were assessed. Interestingly, after immunostaining, researchers found that monocytes derived from older patients (oMs) were more skilled at perfusing the mesh scaffolding and infiltrating cortical organoids – at a rate three times higher than that of young-patient monocytes (yMs). The chemotactic factor MCP-3 was upregulated in oM and organoid co-cultures, which they theorized may explain this observation. Visually, neuron morphology was much poorer in oM-exposed cortical organoids. Higher expression of pro-inflammatory cytokines IL-1B and TNF-α was noted in the oM co-culture, as well as expression of senescence markers p16 and p21. This device demonstrated the differences between young and old immune cells. Although further studies are required, these results imply that including immune cells derived from blood of older populations may be vital to accurately representing age-related neuroinflammatory disorders. One important note is that the system did not recapitulate the BBB, so results may only be relevant to diseases in which this feature is compromised. Future work here could focus on the development of a model containing a barrier feature to increase applicability.

In addition to the immune cells active in central nervous system regulation, the BBB also maintains neural health. Plastic in nature, the BBB enables communication between the central and peripheral nervous systems. Astrocytes and pericytes local to the endothelium assist with maintenance of barrier integrity. The BBB is physically composed of endothelial tight and adherent junctions that induce electrical resistance which limits cross membrane transport, resulting in a highly selective, well-regulated barrier between peripheral circulation and the brain.^71^ Therapeutics targeting the brain are difficult to administer due to this specificity. Immunological models of the BBB are important for a deeper understanding of how immune cells affect the integrity of this neurological feature.

Due to their characteristic role as maintenance cells of the BBB, researchers focus on astrocytes in the immune response in Parkinson's disease.^72^ As the disease progresses, an increase in BBB permeability is observed in Parkinson's patients, as exemplified by an increase in 11C-verapamil uptake in advanced-stage patient brains by Bartels *et al.* in 2008.^73^ Exploring the astrocyte's role in endothelial dysfunction is important to determining novel cellular targets for treatment.

Meena *et al.* developed a dynamic model to observe the aforementioned interaction in 2022.^74^ This pump-based model contained human cerebral microvascular endothelial cells and primary astrocytes, plated on either side of a coated permeable membrane. Custom slides with removable micropore membranes were fabricated, allowing for cell seeding and exposure to continuously flowing media on both sides of the membrane. The flow was maintained at a shear stress rate like that of *in vivo* blood flow. Researchers observed a 30-fold increase in TEER values in the dynamic model, as well as a higher expression of ZO-1 and claudin and occludin genes, all important for tight junction formation. Perhaps most importantly, the permeability of the dynamic model to FITC-dextran was much lower, indicating the development of a functional barrier, representative of the *in vivo* blood–brain interface. Another interesting product of this model was endothelial cell growth in the same direction as medium flow; the development of this atheroprotective feature indicated higher applicability of this model to the human brain, which displays similar growth patterns. This model helped identify the importance of flow and resultant shear stress in development of *in vitro* models of the BBB and may be useful to deducing the permeability of novel therapeutics across the BBB. Future improvements to this model could include adding BBB supportive cells such as pericytes or culturing disease phenotype cells to assess permeability differences compared to healthy ones.

In a system capable of modeling the diseased BBB, researchers created a system composed of iPSC-derived BMEC-like cells, iPSC-derived astrocytes and human primary pericytes.^75^ The model consisted of three lanes – the top lane plated with the iPSC-derived endothelial cells, the middle composed of a semi-rigid ECM gel to allow for bidirectional diffusion and the bottom layer seeded with astrocytes and pericytes. Researchers compared endothelial barrier formation between cells iPSC-derived from Parkinson's patients and healthy controls. With use of healthy control cells in the system, 6 days *in vitro* allowed for formation of an endothelial vessel with junctions that restricted a 4.4 kDa dye, dextran–TMRE, as well as other migratory proteins such as IgG across the border. Pericytes alone were not sufficient for barrier formation – astrocyte presence was paramount to production of this feature, indicating the pivotal role of astrocytes in barrier formation. The use of astrocytes derived from Parkinson's patients resulted in an increased leakage of the same migratory molecules previously restricted in the control model. An upregulation of the inflammatory cytokines IL-6 and CXCL8 and adhesion molecules VCAM and ICAM was noted in the mutant model, providing a potential explanation for the increased barrier permeability. To explore the signaling cascade responsible for this pathology, the researchers performed computational analysis that indicated the MEK/ERK pathways may contribute to the inflammatory response. Pharmacological inhibition of the MEK pathway resulted in a restoration of barrier integrity and reduction of inflammatory secretions in the mutant model, implicating MEK's role in astrocyte activity and resultant BBB dysfunction. This successful pharmacological inhibition provided evidence that the model's response to drug treatment was functional and implies potential for novel therapeutic development.

As MPS continue to garner support for use in modeling and drug development, it remains important to create models that accurately reflect *in vivo* interactions. The current trend toward more frequent use of human cells is an important improvement over past models that utilized rat and mouse cells. Use of human cells gives pertinent insight into a disease environment, providing more physiologically relevant data than that of animal-based MPS models. Ultimately, human-based models will increase the perceived reliability and applicability of microphysiological systems, ideally leading to a higher level of adoption. Inclusion of human innate immunity components in central nervous system models is especially critical for reliability, since innate cells can greatly contribute to disease development.

The inclusion of multiple immune cell types in a single model is an important consideration for future models, as immune components secrete factors that may affect the other immune cells present. For example, an increased risk of mortality in older populations has been associated with elevated levels of IL-6, TNF-α, IFN-gamma and IL-1; these are produced by a variety of immune cells including but not limited to monocytes, macrophages, dendritic cells and natural killer cells.^67^ Combining these cell types into a single model may be essential for creating accurate inflammatory models of aging. Development of Alzheimer's disease has been theorized to be spurred by IL-1, which is released by monocytes, macrophages, and epithelial cells, among others. As discussed previously, microglia are also known to have an inflammatory role in AD.^47^ Devising a central nervous system model that includes neurons, endothelial cells, microglia, and other relevant immune cells could provide novel information about how these cells and their immune outputs contribute to Alzheimer's. Designing a multicellular model that considers the complete picture of a disease state is crucial for successful, valid testing of compounds that target a specific disease mechanism.

## The innate immune system and metabolic tissues

### The gut

The gastrointestinal tract was discussed previously in the epithelial section, yet we will expand the discussion further in relation to its digestive role. The gut does not just regulate immune response to resident microbes; being the primary location of nutrient absorption in our body, the gut must also process and regulate response to nutrient consumption. One of the more common diseases triggered by a fault in the innate immune system are allergies, more specifically food allergies. Not to be confused with food intolerance, which arises from non-immune factors, food allergy is defined as “a pathological reaction of the immune system triggered by the ingestion of a food protein antigen”.^76^ Depending on the individual, exposure to varying amounts of allergenic foods can trigger clinical symptoms (ranging from mild to severe) such as airway inflammation and gastrointestinal disorders.^77^

In 2013 researchers Ramadan *et al.* investigated the multi-functional components of the gut by constructing a model that featured nutritional transport across gut epithelium and immunomodulation in response to nutrient absorption.^78^ This platform, called the NutriChip, was a co-culture system consisting of a confluent epithelial layer of Caco-2 cells and the monocytic cell line U937 that were separated by a porous polyester membrane. The system was manufactured to allow for absorption across the membrane in hopes that an anti-inflammatory response to pathogens would be engaged yet an immunotolerant reaction would occur upon the recognition of harmless nutritional intake.

Allergic reactions are an IgE-mediated immune response. Individuals who experience subsequent exposure to a food allergen after already experiencing an initial immune response experience a rapid onset of allergic reaction symptoms due to IgE-mediated degranulation of innate immune effector cells, such as mast cells and basophils. The attachment of food allergen-derived epitopes to IgE molecules causes epitope-specific cross-linking between IgE-bound receptors, which leads to the release of histamine and the other inflammatory mediators from the effector cells involved in the immediate phase of the allergic reaction. Following this prompt response, a combination of the production of platelet activating factor, leukotrienes, and cytokines such as IL-3, IL-4, and IL-5 maintain the inflammatory response of the allergic reaction.^79^ Many of the current models that study allergies are immunotherapy-induced desensitization models. Allergen-specific immunotherapy (AIT) is a specific, desensitizing treatment that has been used for the past century.^80^ Recently, desensitizing treatments have become a focus point for prevention strategies related to diseases that form due to immune system dysfunction, such as food allergies.^81^ Modeling this immunotherapy in such a system could lead to many positive outcomes such as less drug usage, decreased disease severity, and prevention of future sensitizations to other allergens.^82^

Inflammatory bowel diseases (IBD), which are chronic diseases of the gastrointestinal (GI) tract that typically arise with the correct combination of a high-risk genotype and applicable environmental factors, can be presented phenotypically as either ulcerative colitis (UC) or Crohn's disease (CD). Indeterminate colitis is a term that was coined due to the overlap in clinical presentations, colonoscopy findings, and histopathological features of both UC and CD.^83^ Both the innate and adaptive immune systems play a significant role in the pathogenesis of IBDs. The GI tract is considered highly immunogenic due to the considerable number of immune cells present and is protected from pathogens due to the gut microbiome living in harmony with the immune system. The gut microbiome maintains its tolerance thanks to the homeostasis of the intestinal microbiota, gut epithelial cells, stromal cells of the intestines, antigen-presenting cells (DCs and macrophages), and inflammatory cells (lymphocytes and neutrophils).^84^ Any sort of dysbiosis in the gut microbiome that would cause a dysregulated response by any of these cell types can shift the immune tolerance, which can lead to the development of IBDs.^85^

With the preceding autoimmune diseases, genetics also play a role in the development of UC and CD. In about 20% of CD cases, there is a genetic alteration of the gene coding for nucleotide-binding oligomerization domain 2 (NOD2). This mutation is associated with a decrease in response to bacterial lipopolysaccharides, which causes more Gram-negative bacteria to survive and transpose itself into the bowel epithelium, inducing inflammation.^86^ A different mutation can affect the alleles coding for fructosyltransferase-2, preventing this enzyme from secreting into the intestinal tract which can cause an increased risk for dysbiosis of the gut microbiome.^87^ Another mutation within the IL-10 receptor causes dysfunction within the IL-10 signaling pathway, which has been associated with childhood onset IBD.^88^ Environmental factors such as social stress, smoking, diet, drugs, and even psychological health are considered risk factors for IBDs, as they are for other autoimmune diseases as well.

The work by Maurer *et al.*, mentioned previously in this review, helped to provide proof that disturbances within the microbiota of the gut can cause an inflammatory response, as seen in inflammatory bowel diseases. Kim *et al.* also designed a gut-on-a-chip model that simulates the characteristic inflammation of IBDs.^89^ To replicate the chronically inflamed environment present in IBDs, isolated human PBMCs were introduced into a lower channel of the device. Due to their nature of carrying a variety of innate immune cells, such as monocytes and granulocytes, these are a well-rounded cell type to use when assessing inflammation in the gut. Upon the addition of PBMCs into the device, the system was also exposed to LPS to observe the immune reaction to a disturbance in the normal microbiota of the gut cells within the model. The response consisted of a decrease in barrier integrity (a characteristic also observed in the gut model produced by Maurer *et al.*), as well as destruction of the intestinal villi, another characteristic present in IBDs.

### The pancreas

**Fig. 4:**
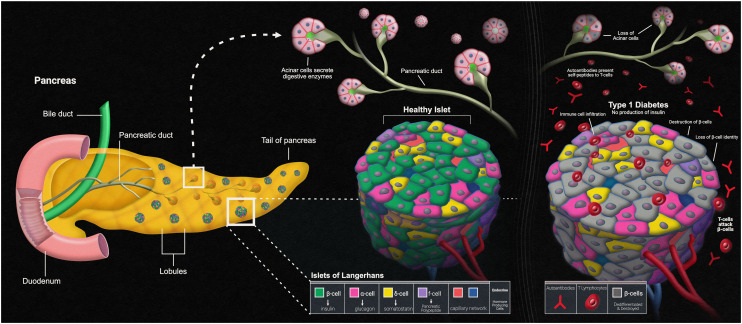
Pancreatic islets and type-1 diabetes.

Another disease that can present itself in the human body due to errors within the innate immune system is type 1 diabetes mellitus (T1D). The main characteristic of T1D is lack of insulin production caused by the T-cell-mediated autoimmune destruction of the pancreatic beta cells, which is based on genetic susceptibility and further triggered by environmental factors, much like other autoimmune diseases.^90^ The main genetic component responsible for affecting the beta cells within the islets of the pancreas is the human leukocyte antigen (HLA) locus, with more than 90% of diagnosed patients possessing DR3 and/or DR4 HLA-DRB1 class II alleles, compared to a far fewer frequency of these alleles in those without T1D.^91^ The presentation of self-peptides to T-cells can start an autoimmune response that affects the pancreatic beta cells. Researchers have discovered that the beta cells essentially go through an “identity crisis” when the autoimmune response begins (Fig. 4). Instead of the cells going through apoptosis, like most other cells in autoimmune diseases, beta cells begin to lose their functional mass by dedifferentiating into a diabetic state. Dedifferentiation consists of the beta cells regressing to a precursor-like state, losing the key components needed for proper insulin secretion.^92^ There is evidence to further indicate that upon beta cell dedifferentiation, the cells could be going through trans-differentiation into the other pancreatic endocrine cell types, alpha and/or delta cells, which have their own separate functions.^93^ To fully understand the autoimmune aspects of T1D, organ-on-a-chip models containing a pancreatic cell component are and will continue to be of vital use.

Pancreas-on-a-chip models (PoC models) seem to be the new way that researchers are studying T1D. Over a decade ago, Mohammed *et al.* created their own microfluidic pancreas-on-a-chip device for the sole purpose of characterizing pancreatic islet cells post-isolation. This is a PDMS-based device designed to allow peristaltic pumps to circulate both culture medium and glucose solutions to perform various tests such as intracellular Ca^2+^ measurements upon glucose challenge and dynamic insulin secretion based on glucose responsiveness.^94^ Since the autoimmune aspect of T1D is mediated by T-cells, the autoimmune status of this disease relies on the presence of islet autoantibodies. T1D can be screened for by carrying out two different types of assays. An enzyme-linked immunosorbent assay (ELISA) has been used to detect interferon-γ-secreting T-cells that respond to both C-peptide and glutamic acid decarboxylase-65 antigens, while a radioligand assay has been used to detect autoantibodies against the different antigens of glutamic acid decarboxylase, islet antigen-2, and zinc transporter-8.^95^ Utilizing this model, once the islet cells were characterized post-isolation, these two different immunoassays could have been run to detect the autoantibodies against the varying antigens, but Mohammed *et al.* did not have all the information regarding these antigens back then. This model serves as a baseline for other, more complex models that have been developed to study T1D using immunoassays, with these PoC models adding automated aspects and/or incorporating other organs that play a role in the pathophysiology of the disease.

The focus of most PoC models is to observe insulin secretion in response to glucose stimulation, as this information can further be used to assess beta cell function within the pancreatic islets. A PoC model designed by Lomasney *et al.* applies capillary electrophoresis-based immunoassays (CEIA) in a glass chip within their platform to detect insulin. This chip is also capable of flow switching, which switches the glucose concentration within the system every five seconds to detect the response of insulin secretion to changing glucose levels.^96^

Another, more advanced PDMS-based PoC model was created by Bauer *et al.* with the purpose of the functional coupling of human pancreatic islet microtissues and liver spheroids composed of HepaRG cells and primary human stellate cells. By co-culturing these two cell types in a microfluidic chip, they were able to demonstrate insulin release by the islets in response to glucose and the glucose uptake by the liver organoids, making the model useful for studying the effects of different Diabetes medications on glucose regulation.^97^ Like many others, including Mohammed *et al.* and Lomasney *et al.*, this model by Bauer *et al.* utilized intracellular Ca^2+^ oscillation monitoring and CEIA to monitor the insulin secretion from the islets. Many PoC platforms have advanced designs but still require traditional sampling followed by ELISA quantification for analysis.

A fully automated PoC platform with an integrated “on chip” insulin immunoassay was designed by Glieberman *et al.*^98^ Within this platform the islets receive automated glucose stimulation, with the chip containing integrated fluorescent glucose tracking along with the built-in insulin immunoassay that allows glucose stimulation tracking and continuous insulin measurement of the islets inside the chip. Among the various MPS focused on diabetes research, all allow for the flow of specific immune cells over human pancreatic islets. Immunofluorescent imaging provides a way to monitor cell phenotypes within the islets in response to any diabetogenic stimuli.^99^

As previously discussed, most of the existing PoC models that currently exist focus on the characterization of the pancreatic cells involved in T1D and the insulin release from the islets in response to glucose, which is a great starting point for diabetes research. Further models should really focus on integrating even more immune components so that the specifics regarding the autoimmune aspect of T1D can also be studied, such as the HLA locus and beta cell dedifferentiation. These models could include a mix of a pancreatic organ construct, a gut organ construct, a liver organ construct, and free-floating immune cells to represent a diabetic state in an MPS model. In this model, the autoimmune aspect of beta cell dedifferentiation in the pancreas could be monitored over time. Since T1D influences the body's metabolic system, including the liver and gut constructs could be beneficial to see if there are any autoimmune effects on these cell types as well. Performing medium collections and running various assays such as ELISAs would make it possible to see the diverse types of immune cells that illicit a response to diabetic stimuli within the model. Combining all these aspects together could really help to tell the story of autoimmunity in T1D.

### The liver

Although the primary functions of the liver are metabolism and protein synthesis, a large population of immune cells localized in the liver play a key role in the body's innate immunity. Receiving arterial blood supply from systemic circulation *via* the hepatic artery, and venous outflow of the intestine *via* the portal vein, the liver processes nutrients from this blood and filters out toxins. This venous blood then drains from the liver *via* the hepatic vein and is delivered to the inferior vena cava. Unlike other organs, the liver's capillary network is composed of blood-filled sinusoids surrounding the cells. Of the total blood received from both inputs, roughly 75–80% is accounted for from the portal vein, directly exposing the liver to gut components such as bacteria, toxins, and antigens.^3^

As the largest internal organ, the architectural design of the liver creates a unique oxygen gradient that allows a significant, varied cell population. Hepatocytes, or parenchymal cells, account for 70–80% of liver volume, while non parenchymal cells (NPCs) such as liver sinusoidal cells (LSECs), Kupffer cells (KCs), and hepatic stellate cells (HSCs), account for only 5–6%. The remaining volume is composed of extracellular spaces: bile canaliculi, sinusoid, and Disse. Dividing the architecture of the liver provides five hepatic lobules: the portal triad, composed of the hepatic artery, portal vein, and bile duct, the linear cords of hepatocytes between sinusoids, and the central vein. This unique architecture creates a division of labour between cells and cell types, each playing a different role dependent on their location within the liver and contributing to the body's immune response either directly or indirectly. This environment also holds one of the largest immune cell populations of leukocytes and phagocytes, all strategically located throughout.

Hepatocytes (HCs) are main producers of secreted PRRs such as complements, pentraxins, peptidoglycan-recognition receptors, and lipid transferases.^3,100^ Hepatocytes synthesize these proteins and secrete them into the bloodstream to protect the body against local and systemic infection. This ability to secrete PRRs allows hepatocytes to play a key role in regulating the immune response. Bouwman *et al.* showed that transplantation of a liver with a genetic predisposition to lower levels of secreted PRRs introduced a higher risk of infection for the patient post transplantation; thus, proving the importance of the role hepatocytes play in the body's innate immune response.^101^

**Fig. 5:**
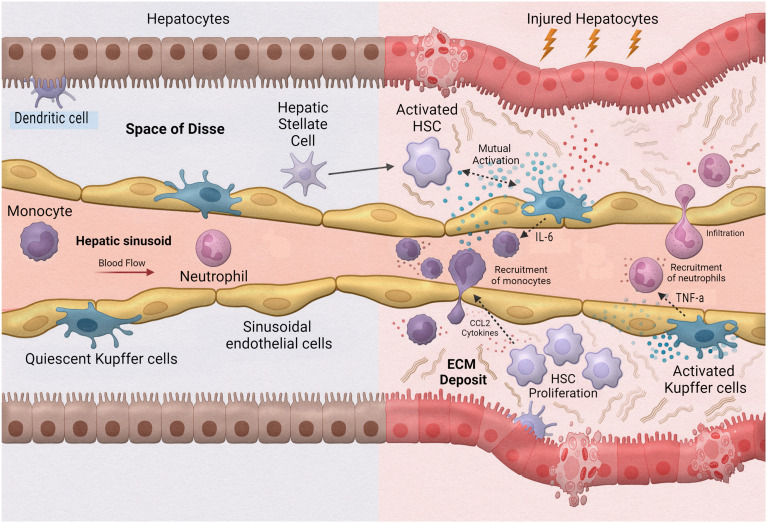
Innate immune cells in liver inflammation.

One of the leading causes of liver transplants in the United States is non-alcoholic fatty liver disease (NAFLD), a broad spectrum of conditions ranging from hepatic steatosis to non-alcoholic steatosis, fibrotic non-alcoholic steatohepatitis (NASH), and liver cirrhosis.^102^ As such, the implementation of MPS to study this disease is extremely valuable. Activation during the innate immune response is a fundamental aspect of hepatic inflammation in NAFLD/NASH (Fig. 5). During homeostasis, or physiological equilibrium, Kupffer cells, hepatic stellate cells, and liver sinusoidal endothelial cells remain in a quiescent state with only a few monocyte-derived macrophages and neutrophils recruited from the sinusoids. Chronic hepatic liver injury results in the activation of Kupffer cells and hepatic stellate cells causing inflammation and fibrosis. Activated Kupffer cells release inflammatory cytokines and chemokines, such as TNF-α and IL-6 to initiate inflammation. These activated Kupffer cells can then cause activation in liver sinusoidal endothelial cells as well as simulate recruitment of neutrophils and macrophages. Cytokines released by Kupffer cells and recruited monocyte-derived macrophages result in activation of hepatic stellate cells. Hepatic stellate cells also respond to damage associated molecular patterns (DAMPs) and pathogen-associated molecular patterns (PAMPs) which are increased in NAFLD/NASH. Activated hepatic stellate cells, Kupffer cells, and innate immune cells interact causing trans-differentiation into proliferative myofibroblasts which produce excess extracellular matrix, or fibrosis.

In a 3D *in vitro* model of NAFLD, Kostrzewski *et al.* (2017) evaluated the effects of fat loading on primary human hepatocytes.^103^ Their results found that hepatocytes exposed to fat-containing medium accumulated three times more fat than cells in lean medium. The results also showed an increase in expression of genes associated with NAFLD such as insulin-like growth factor-binding protein 1, fatty acid-binding protein 3, and CYP7A1. Wen *et al.* found treatment of free fatty acids to HepaRG cells in a MPS resulted in induced lipid accumulation in the cells as well as an increase in apoptotic cells.^104^ The accumulation of fat in the cells induces hepatocellular stress, injury, and death. This can lead to fibrogenesis and genomic instability making the liver susceptible to cirrhosis hepatocellular carcinoma.^105^ Slaughter *et al.* (2021) generated a 2-organ model consisting of hepatocytes and adipocytes to evaluate the adverse effects fat tissue imposes on the liver in the case of NAFLD.^106^ In this study they found that exposure to pro-inflammatory markers in this co-culture system resulted in hepatocyte steatosis. However, this did not occur in a monoculture of hepatocytes alone. This study demonstrated that inflammation influences adipokine secretion profiles and lipolysis which further impacts the health of hepatocytes which aligns with clinical findings. While these three studies helped to provide a foundation for modelling NAFLD on a chip, there are many other cell types in the liver that are relevant to the pathology of non-alcoholic liver diseases. Freag *et al.* addresses this in a study from 2022 in which they created a NASH-on-a-chip model using a coculture (HCs, KCs, and HSCs) hydrogel 3D scaffold with LSECs in a blood vessel mimetic endothelialisation inlet channel.^107^ Using free fatty acids as lipotoxic stressors and LPS to trigger disease progression, Freag *et al.* found a time-dependent response of an accumulation of lipids in the cytoplasm of HCs resulting in apoptosis. Injury of HCs by lipotoxic stressors causes hepatocellular ballooning which releases extracellular vesicles initiating HSC activation and stimulating innate immune cells.

Liver sinusoidal endothelial cells (LSECs) are strategically localized in the lining of the vascular bed. In this location the cells are continuously exposed to both arterial and venous blood, allowing them to remove and recycle lipids and proteins. LSECs are considered gatekeepers of liver homeostasis. This is partially due to their ability to activate KCs and HSCs to promote fibrogenesis as well as promote the synthesis of endogenous cholesterol in a disease state of NAFLD.^108^ However, in a healthy liver, these cells display anti-inflammatory and anti-fibrogenic characteristics by preventing cell activation and regulating vasodilation.^109^ Additionally, LSECs have a high endocytic capacity, higher than any other cell in the body, due to their expression of endocytic receptors (*e.g.*, macrophage mannose receptor, scavenger receptors, Fc gamma-receptor IIb2 (CD32B)). Some of the well-known substances LSECs endocytose include hyaluronan, heparin, collagen α chains, IgG immune complexes, acetylated low density lipoprotein (ac-LDL), mannan, and ovalbumin (OVA).^108,110^ LSECs along with the help of KCs scavenge and clear the blood of viruses, advanced glycation end products, and modified LDL cholesterol.^110^ Since the liver is constantly exposed to food and microbial antigens *via* the gut, an important balancing act of the immune response is necessary for maintenance. This exposure first occurs in the sinusoids – therefore, KCs and LSECs play a key role in this regulation by taking up and eliminating antigens and determining an immune response. For example, chronic exposure of KCs and LSECs to certain antigens, such as LPS, puts KCs and LSECs into a refractory state, preventing the liver from being in a constant state of inflammation due to the presence of these gut bacterial products.^111^ Differing from other antigen presenting cells, LSECs have cell-specific responses which contribute to the tolerogenic environment of the liver. These cell-specific responses are due in large part to the high expression of scavenger receptors by LSECs. LSECs ability to express receptors allows the induction of pro-inflammatory and anti-inflammatory signals and direct interaction with toll-like receptors. Members of the C-type lectin family, a type of scavenger receptor found on LSECs, are directly involved in the uptake of viral particles such as the hepatitis C virus, Ebola virus, HIV, and coronavirus.^108,111^ With endocytic receptors that bind multiple viruses, LSECs perform a decisive role in the body's response to viral infections and help mediate the removal of blood-borne viruses. In a mouse infection study, Ganesan *et al.* shows the ability of LSECs to clear the body of adenovirus within minutes with about 90% of the virus found in LSECs and around 10% in KCs.

In a model created by Li *et al.* upon activation of LSECs by molecular driver's characteristic of disease progression of NAFLD conditions (LPS, EGF, TNF-β) a significant increase in polymorphonuclear neutrophils (PMNs) bound to activated LSECs can be seen.^112^ These PMNs migrate to the hepatic chamber from the vascular channel in response to the pro-inflammatory molecular drivers after binding to the activated LSECs. To examine the effects of hepatic non-parenchymal cells following alcohol exposure in alcoholic liver disease (ALD), Deng *et al.* 2019 created a MPS that incorporated HepG2 cells, HSCs, LSECs, and KCs and evaluated the biomarkers associated with this disease.^113^ This study found that exposure to ethanol damages the tight junction of LSECs and leads to the activation of HSCs.

The largest population of macrophages, around 80% of the body's total, inhabit the liver. These resident hepatic macrophages, or KCs, play a key role in innate immunity and maintaining homeostasis. KC's primary function is to act as a scavenger cell to provide a firewall for the liver for clearance of bacteria and microbes that enter the bloodstream *via* the gut.^114^ A well-known ability of KCs is overtaking bacteria and responding to antigens, such as LPS and interferon γ.^115,116^ Luster *et al.* (1994) demonstrated that KCs in response to LPS release the peptide mediators, TNF-α and IL-1.^117^ KCs take up endotoxins and phagocytose bacteria delivered through the portal vein through the expression of TLR4 and CD14.^116^ In acute and chronic liver disease, KCs produce TNF-α and reactive oxygen intermediates.^115,118^

Nawroth *et al.* modelled ALD using a Liver-Chip coculture composed of primary human hepatocytes, primary liver sinusoidal endothelial cells, and KCs.^119^ Exposure to human relevant blood alcohol concentrations of ethanol for 48 h established ALD markers such as intracellular accumulation of lipids, development of oxidative stress, and cholesterol synthesis dysregulation. Alcohol consumption negatively affects intestinal barrier function which results in increased permeability to endotoxins such as LPS. This study also included the effects of ethanol + LPS to determine if this combination would worsen steatosis and oxidative stress. A dose-dependent increase in mitochondrial reactive oxygen species was observed in ethanol exposure alone and these increases were compounded with the addition of LPS exposure. In addition, inflammatory cytokines such as TNF-α and IL-6, with which KCs are main producers of, were significantly increased with treatment of LPS and co-treatment of ethanol + LPS.

Kostrzewski *et al.* (2020) developed an *in vitro* human liver model using a three-dimensional MPS with a coculture of microtissues containing primary human hepatocytes, KCs, and stellate cells to represent NASH.^120^ NASH is a severe form of NAFLD caused by a buildup of fat in the liver that can eventually lead to cirrhosis and liver cancer. In the NASH co-culture system, changes in gene expression were observed in 25 genes including genes associated with inflammation (*e.g.*, IL-6, TNF-α). Additionally, they evaluated how fat and repeat dosing of LPS affects the phenotype within the NASH model. Fat loading of cells and more significantly the LPS dosing increased the secretion of IL-6, and TNF-α. Concluding that fat and LPS promoted the disease state by significantly enhancing the inflammatory response in this model. Li *et al.* used a glass-based zonated and vascularized human liver MPS model to evaluate the effects of molecular drivers (LPS, EGF, TNF-β) associated with NAFLD/NASH.^112^ The study found the induction of LPS resulted in the release of TNF-α from the KCs.

HSCs reside in the space of Disse, between parenchymal cells and LSECs. The previous decades of information on HSCs describe these cells as dormant, or quiescent, and mainly responsible for the storage of vitamin A, maintenance of hepatic architecture, and synthesis of polypeptide mediators which potentially benefit liver regeneration or recovery. However, more recent studies show HSCs also assist in the immune response by acting as antigen presenting cells, expressing PRRs, responding to damage associated molecular patterns, and through interacting with immune cells by modulating their activity or promoting differentiation. While HSCs remain quiescent in a healthy liver, these cells are activated through inflammatory pathways and induce a fibrotic response. Studies suggest HSCs express certain toll-like receptors as well as increase differentiation and stores of regulatory T cells.^121^ By expressing retinoic acid, HSCs also directly interact with NK cells, NKT cells, and T cells.^122,123^

Using a quadruple-cell Liver-Chip model, Jang *et al.* reproduced drug-induced liver injury (DILI) responses to clinically relevant drug concentrations which generated results that closely represented those found in nonclinical as well as human clinical trials.^124^ While most *in vitro* studies are not able to examine the mechanisms behind DILI or relevant endpoints, this study found various phenotypes of DILI such as hepatocellular injury, cholestasis, steatosis, KC depletion, and stellate cell activation.

NASH causes HSCs to differentiate into myofibroblast-like cells that cause fibrosis, predisposing patients to cirrhosis and liver cancer. Mentioned previously, the study from Kostrzewski *et al.* (2020)^120^ also evaluated the effects of a mutant protein found in HSCs which increases an activation of these cells, causing these cells to become more pro-inflammatory, and increasing their lipid content. They confirmed that this mutant type can enhance the overall NASH disease state. Another study evaluating NASH, recreated the early stages of this disease using a MPS design that integrated hepatic sinusoidal flow, transport, and lipotoxic stress risk factors (glucose, insulin, free fatty acids) with primary human hepatocytes, HSCs, and KCs.^125^ They found fibro-genic activation markers such as TGF-β, ECM gene expression, and HSC activation, increased with lipotoxic conditions. Inflammatory analyte secretion (*e.g.*, IL-6, IL-8, alanine aminotransferase) also increased with these changes. In the study by Li *et al.*, the exposure of the molecular drivers (LPS, EGF, TNF-β) related to NASH/NAFLD increased α-SMA expression which indicates the activation of stellate cells into proliferating, collagen-depositing myofibroblasts.^112^

As discussed, the complex, unique design of the liver creates an oxygen gradient and a division of labour between the main cell types that is difficult to imitate *in vitro*. In 2017, researchers Lee-Montiel *et al.* recognized this complexity of the liver and generated a Liver Acinus MicroPhysiological System (LAMPS) to model specific niches of oxygen gradients within the liver and the resulting effects on cell physiology.^126^ This impressive model incorporated primary human hepatocytes, human dermal microvascular endothelial cells (HMVEC-D), THP-1 cell line to represent KCs, and the LX-2 human stellate cell line. This study found that oxygen concentrations have a substantial impact on the physiology of the liver. Their model was continuously exposed to higher oxygen concentrations to represent zone 1 of the liver, while a second system representing zone 3 of the liver was perfused with lower oxygen levels. This study found that in the zone 1 model yielded higher levels of oxidative phosphorylation, glucose, urea, and albumin. The zone 3 liver model yielded higher levels of α1AT, CYP2E1, and lipid droplets. This increase in lipid droplets such that zone 3 hepatocytes are more susceptible to steatosis. Further, when each of these models were exposed to acetaminophen, zone 3 hepatocytes yielded higher levels of LDH, which correlates to *in vivo* studies that zone 3 liver cells are more susceptible to acetaminophen toxicity. While this model did not directly assess immune response in this study, it is helpful to understanding the effects of oxygen zonation with a model that incorporates immune cells. This offers a platform to study an array of liver diseases that illicit an inflammatory immune response.

A limiting factor in this field is the lack of *in vitro* models that accurately reproduce the physiology of the human liver, especially through the use of primary human donor cells as opposed to cell lines. Future endeavours will require advancements in design and technology as well as in cell composition (organoids, monolayers, cocultures) to better emulate liver architecture and function. As understanding of the liver and its function in immunity continues, and as technology expands, creating a MPS design that reliably represents the liver is attainable.

## Conclusion

The innate immune system has many roles in regulating the organ systems within the human body. Many organ-on-a-chip models focus on modelling of one organ system or cellular interaction, and therefore may not be ideal in modelling systemic diseases that affect multiple organs. Progress has been made to integrate these individual immune components into more complex multi-organ microfluidic systems.

In 2016, Miller & Shuler described a 14 compartment MPS that allowed for combination of 13 different cell lines, including spleen cells as an immune component.^127^ Although immune output was not quantified in the initial report, this model could be used in future studies to illustrate the interactions of the immune system with other organs. Recently, researchers Rupar *et al.* fabricated a microfluidic model containing primary human erythrocytes, hepatocytes, endothelial cells, and splenocytes in the development of the Malaria-on-a-chip model.^128^ This proof-of-concept model enabled future monitoring of immune organ response to parasite infection and reaction to treatment administration. Using a multi-organ MPS containing innate and adaptive immune cells, Trapecar *et al.* assessed immune-metabolic crosstalk between the gut, liver, and brain in a Parkinson's disease model.^129^ The innate immune system was represented by preparing the gut MPS with seeded human monocyte derived DCs and KCs as the innate component of the liver. Adaptive immunity was also included by using culture medium containing CD4+ regulatory T_H_17 cells. Inclusion of both innate and adaptive immune components resulted in cytokine and chemokine values like those of human plasma. Innate as well as adaptive immune components must be considered for the most proper *in vitro* imitations of the human body.

Our understanding of immune responses and organ interactions have grown significantly because of multi-organ MPS development. However, there is still room for growth in this field, especially as we explore the delicate immune interactions with other immune cells in multiorgan platforms. Current studies have yet to investigate the multifaceted relationship between innate immune cell populations in a human-on-a-chip platform, such as interactions between neutrophils, macrophages, and DCs.^100^ Modelling crosstalk between these cell types in a human-on-a-chip platform is important for determining immune cooperation and how it is altered by presence or absence of different cells. Expanding further, utilizing MPS to study autoimmune diseases would have many benefits. There would be a reduction in harm to mice or other animals required to be infected with such diseases to study the mechanisms of the dysfunctional immune system. Another benefit is that systems can be designed with many different organ constructs, and different diseases that have overlap in the affected organs can be studied simultaneously, allowing for further connections of mechanisms of action between organs and immunity. Ultimately, including innate components in multi-organ MPS may help elucidate novel molecular pathways active in disease progression. This focus is critical for continued progress in identifying targets for future drug development.

## Abbreviations

ALI: Air–liquid interface
ALD: Alcohol-associated liver disease
APC: Antigen presenting cell
BBB: Blood–brain barrier
CEIA: Capillary electrophoresis-based immunoassays
CNS: Central nervous system
COPD: Chronic obstructive pulmonary disease
CD: Crohn's disease
DC: Dendritic cell
DILI: Drug-induced liver injury
ELISA: Enzyme-linked immunosorbent assay
GI: Gastrointestinal
HSC: Hepatic stellate cell
hBM: Human bone marrow
HLA: Human leukocyte antigen
hLN: Human lymph nodes
HMVEC-D: Human dermal microvasculature endothelial cells
IBD: Irritable bowel disease
KC: Kupffer cells
LAMPS: Liver acinus microphysiological system
LTB4: Leukotriene B4
LPS: Lipopolysaccharide
LSECs: Liver sinusoidal endothelial cells
MSCs: Mesenchymal stromal cells
MAMP: Microbial associated molecular pattern
MPS: Microphysiological systems
NK: Natural killer
NETs: Neutrophil extracellular traps
NAFLD: Non-alcoholic fatty liver disease
NASH: Non-alcoholic steatohepatitis
PAMP: Pathogen-associated molecular pattern
PRR: Pattern-recognition receptor
PBMCs: Peripheral blood mononuclear cells
SDS: Sodium dodecyl sulfate
TNF-α: Tumor Necrosis Factor alpha
T1D: Type 1 diabetes mellitus
UC: Ulcerative colitis

## Author contributions

MJR, HH, SR, and BB were all equally responsible for writing the critical review. ST was responsible for generating all figures in this critical review. MJR and JJH were responsible for reviewing and editing the critical review.

## Conflicts of interest

There are no conflicts to declare.
